# Assessment of intramyocardial hemorrhage in acute reperfused myocardial infarction using 7.0T

**DOI:** 10.1186/1532-429X-17-S1-O76

**Published:** 2015-02-03

**Authors:** Wei Chen, Bing Zhang, Fabao Gao, Jie Zheng, Rui Xia, Yushu Chen, Chunhua Wang

**Affiliations:** 1 Radiology Department, West China Hospital, Sichuan University, Chengdu, China; 2 Radiology Department, 1st Affiliated Hospital of Chongqing Medical University, Chongqing, China; 3 Mallinckrodt Institute of Radiology, Washington University School of Medicine, St. Louis, MO, USA; 4 Radiology Department, 1st Affiliated Hospital of Kunming Medical University, Kunming, China

## Background

Intramyocardial hemorrhage(IMH) caused by reperfusion after acute myocardial infarction(AMI) is considered to be an important independent predictor of adverse left ventricular remodeling and clinic outcomes. We aim to validate whether CMR T2 mapping has a high diagnostic accuracy for IMH and if it is able to quantify IMH.

## Methods

10 Sprague Dawley rats (female, 250-300g) underwent ligation of the left anterior descending or circumflex coronary arteries for 60 minutes, followed by reperfusion for 48 hours. The rats were then scanned at 7.0T MR (BRUKER BIOSPEC 70/30, Germany), using T2 mapping(TR/TE=1500ms/10,20,30ms, MTX=192×192, FOV=50×50mm, slice thickness=1.5mm) and LGE(TR/TE=5.2ms/1.8ms, FA=25°, MTX=256×256, FOV=50×50mm, slice thickness=1.5mm).The T2 mapping images were created using a custom-made software, written by Matlab. The T2 values of edema, hemorrhage, and remote myocardium were then calculated using a ImageJ software, along with the determination of presence and size of hemorrhage. All datas were assessed by 2 radiologists who were blinded to the pathology results. The hearts were dissected after the CMR study for pathological analysis. Left ventricular sections of the specimens, matched well with T2 mapping slices, were assessed for hemorrhage and its size. The pathological stains including hematoxylin-eosin and Prussian blue staining were assessed for red blood cells exudation and acute hemorrhage respectively by an experienced pathologist.

## Results

**Figure 1:**
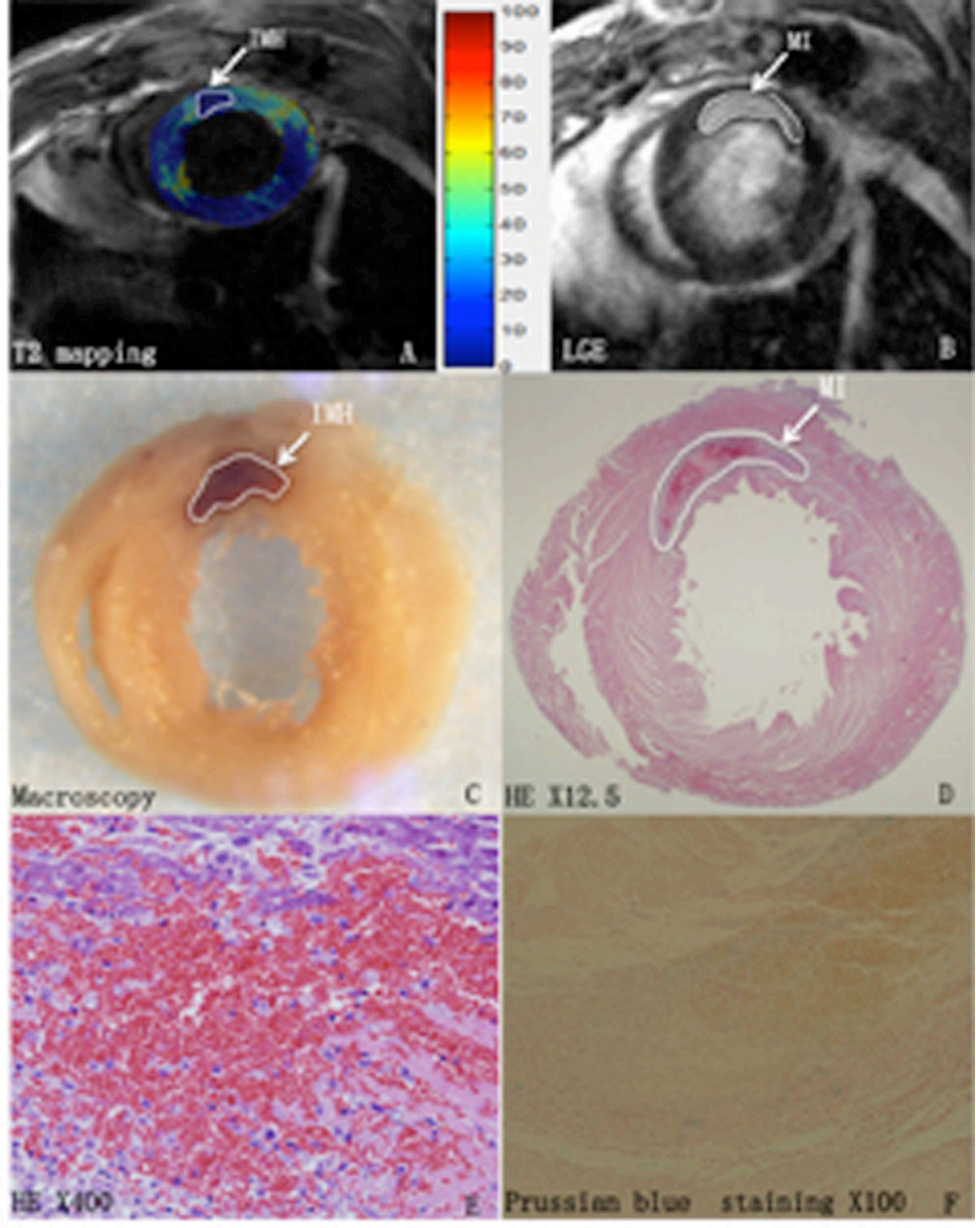
Assessment of Intramyocardial Hemorrhage Using T2 mapping Compared With Pathology

One rat died undergoing CMR. 55 of all 72 pathological sections of left ventricles (LV) showed myocardial infarction that matched well with the hyperintense area in LGE MRI (Figure 1B,D). Myocardial hemorrhage pathologically occurred in 44 sections, corresponded with hypointense area in 41 image slices on T2 mapping (Figure 1A,C,E). Prussian blue staining of all hemorrhagic sections was negative (Figure 1F). 4 slices with hypointense area on T2 mapping showed no hemorrhage on pathological sections. On the section bassis, the sensitivity and specificity for hemorrhage on T2 mapping were 93% and 85% respectively, and the positive and negative predictive values were 91% and 89% respectively (Table 1). The T2 values of edema, hemorrhage, and remote myocardium were 47.2±9.4ms, 27.9±5.2ms, 21.3±4.4ms respectively, and all three values had significant differences (*p*<0.05).The size of hemorrhage calculated on T2 mapping corresponded with that on pathological sections(6.1%±0.02 vs 6.3%±0.02, *p*=0.46).

**Table 1:** Detection of Intramyocardial Hemorrhage Using T2 mapping Compared With Pathology

T2 mapping	Pathology		
	
	Hemorrhage	No hemorrhage	
Hypointense area n=45	41	4	93%, Sensitivity

No hypointense area n=27	3	24	85%, Specificity

	91%, PPV	89%, NPV	

PPV = positive predictive value; NPV = negative predictive value.

## Conclusions

CMR T2 mapping not only has high diagnostic accuracy for IMH in reperfused AMI (even in 48h), but also is capable of quantifying the size of IMH, which may have a potentially significant value in evaluating therapeutic effect and prognosis in patients with AMI after primary percutaneous coronary intervention.

## Funding

This study was supported by The National Natural Science Foundation of China (81130027).

